# Overexpression of circulating MiR-30b-5p identifies advanced breast cancer

**DOI:** 10.1186/s12967-019-02193-y

**Published:** 2019-12-30

**Authors:** Helena Estevão-Pereira, João Lobo, Sofia Salta, Maria Amorim, Paula Lopes, Mariana Cantante, Berta Reis, Luís Antunes, Fernando Castro, Susana Palma de Sousa, Céline S. Gonçalves, Bruno M. Costa, Rui Henrique, Carmen Jerónimo

**Affiliations:** 1 Cancer Biology and Epigenetics Group, IPO Porto Research Center (CI-IPOP), Portuguese Oncology Institute of Porto (IPO Porto), Research Center-LAB 3, F Bdg, 1st floor, Rua Dr António Bernardino de Almeida, 4200-072 Porto, Portugal; 2 Master in Oncology, Institute of Biomedical Sciences Abel Salazar – University of Porto (ICBAS-UP), Porto, Portugal; 3 Department of Pathology, Portuguese Oncology Institute of Porto, Porto, Portugal; 4 Department of Pathology and Molecular Immunology, Institute of Biomedical Sciences Abel Salazar – University of Porto (ICBAS-UP), Porto, Portugal; 5 Department of Laboratory Medicine, Portuguese Oncology Institute of Porto, Porto, Portugal; 6 Department of Epidemiology, Portuguese Oncology Institute of Porto, Porto, Portugal; 7 Department of Medical Oncology, Portuguese Oncology Institute of Porto, Porto, Portugal; 8 Life and Health Sciences Research Institute (ICVS), School of Medicine, University of Minho, Campus de Gualtar, Braga, Portugal; 9 ICVS/3B’s - PT Government Associate Laboratory, Braga/Guimarães, University of Minho, Campus de Gualtar, Braga, Portugal

**Keywords:** Breast cancer, Biomarkers, MicroRNAs, Metastasis

## Abstract

**Background:**

Breast cancer (BrC) remains the leading cause of cancer-related death in women, mainly due to recurrent and/or metastatic events, entailing the need for biomarkers predictive of progression to advanced disease. MicroRNAs hold promise as noninvasive cancer biomarkers due to their inherent stability and resilience in tissues and bodily fluids. There is increasing evidence that specific microRNAs play a functional role at different steps of the metastatic cascade, behaving as signaling mediators to enable the colonization of a specific organ. Herein, we aimed to evaluate the biomarker performance of microRNAs previously reported as associated with prognosis for predicting BrC progression in liquid biopsies.

**Methods:**

Selected microRNAs were assessed using a quantitative reverse transcription-polymerase chain reaction in a testing cohort of formalin-fixed paraffin-embedded primary (n = 16) and metastatic BrC tissues (n = 22). Then, miR-30b-5p and miR-200b-3p were assessed in a validation cohort #1 of formalin-fixed paraffin-embedded primary (n = 82) and metastatic BrC tissues (n = 93), whereas only miR-30b-5p was validated on a validation cohort #2 of liquid biopsies from BrC patients with localized (n = 20) and advanced (n = 25) disease. ROC curve was constructed to evaluate prognostic performance.

**Results:**

MiR-30b-5p was differentially expressed in primary tumors and paired metastatic lesions, with bone metastases displaying significantly higher miR-30b-5p expression levels, paralleling the corresponding primary tumors. Interestingly, patients with advanced disease disclosed increased circulating miR-30b-5p expression compared to patients with localized BrC.

**Conclusions:**

MiR-30b-5p might identify BrC patients at higher risk of disease progression, thus, providing a useful clinical tool for patients’ monitoring, entailing earlier and more effective treatment. Nonetheless, validation in larger multicentric cohorts is mandatory to confirm these findings.

## Background

Breast cancer (BrC) is the second most frequent cancer worldwide and the most commonly diagnosed cancer among women [1]. Despite improvements in early detection and treatment, BrC remains the leading cause of cancer-related death in women, mainly due to the development of recurrent and/or metastatic disease [1, 2]. In fact, at diagnosis, approximately 5% of patients present distant metastases and up to 10–15% of patients develop distant metastases within the first 3 years [3].

Metastatic BrC arises following the proliferation and dissemination of malignant cells from its primary location to distant organs [4]. Metastases often display an organ-specific pattern of spread, with bone constituting the most common site for BrC, followed by lungs, liver, and brain [3]. The development of metastases is the major prognostic factor for BrC patients, as metastatic BrC is incurable in most cases [5]. Thus, adequate patient monitoring, using minimally invasive biomarkers, that might anticipate the diagnosis of progressing disease, might allow for earlier implementation of adequate therapy, improving BrC patients’ survival and quality of life.

MicroRNAs (miRNAs) are endogenous, highly conserved small non-coding RNAs of approximately 22 nucleotides in length involved in genes’ posttranscriptional regulation [6]. Recently, the value of miRNAs as cancer biomarkers has been emphasized, emerging as promising diagnostic, prognostic and predictive biomarkers for BrC [7], including advanced BrC [8, 9]. Moreover, several lines of evidence implicate miRNAs either as promotors or suppressors of BrC metastization, by targeting multiple signaling pathways and important proteins that are major players at different steps of the metastatic process [8, 9].

Specifically, the role of miR-200 family (miR-200f) members in the initial steps of the metastatic cascade, by regulating *ZEB1* and *ZEB2* expression and consequently the *E*-*cadherin* expression has been described by several groups [10, 11]. Likewise, the miR-30 family (miR-30f) members have been previously reported to promote tumor invasion and metastases formation in melanomas [12], whereas in BrC downregulation was implicated in the metastatic process [13, 14]. Conversely, miR-182-5p upregulation was associated with epithelial-like state in BrC cell lines and macrometastases formation in vivo [15].

Nonetheless, we have previously shown that miR-30b-5p, miR-30c-5p, miR-182-5p and miR-200b-3p expression levels associated with patients prognosis in luminal BrC [16].

Thus, we set out a study aiming to assess the biomarker potential of the previously identified miRNAs expression for predicting BrC progression and dissemination in liquid biopsies.

## Methods

### Patients and samples collection

Formalin-fixed paraffin-embedded (FFPE) primary and metastatic tumors available from BrC patients were included in this study: 16 patients for the testing cohort and 82 for the validation cohort #1. Sixteen primary BrC and 22 paired metastases were obtained from the testing cohort and 82 primary BrC and 93 paired metastases were obtained from the validation cohort #1. Relevant clinical data were retrieved from the patients’ charts. All cases were revised by an experienced pathologist, classified according to the most recent 4th edition World Health Organization (WHO) classification, graded according to Bloom and Richardson’s Modified system and staged according to the 7th edition American Joint Committee on Cancer (AJCC) system [17, 18]. Four μm sections were cut from each tissue block and stained with hematoxylin–eosin, which were examined by the pathologist to select the most representative tumor lesion. Tumor areas identified were then macrodissected in 6 consecutive 8 µm sections for tumor cells enrichment (> 80%).

Additionally, peripheral blood samples from 20 patients with localized BrC and 25 patients with advanced BrC were collected at the Portuguese Oncology Institute of Porto (IPO Porto) after informed patient consent (validation cohort #2). Briefly, peripheral blood was collected into EDTA-containing tubes and centrifuged at 2000 rpm for 10 min at 4 °C. Plasma was immediately separated, aliquoted into 1.5 mL tubes and properly stored at − 80 °C until further use.

This study was approved by the institutional ethics committee (Comissão de Ética para a Saúde, CES-IPOFG-EPE 019/08 and CES 120/015) and sample collection was performed in accordance with the Declaration of Helsinki. Informed consent was obtained from all individual participants included in the study.

### RNA extraction

RNA extraction from FFPE tissues was performed using a commercially available extraction kit (FFPE RNA/DNA Purification Plus Kit, Norgen Biotek, Thorold, Canada) in accordance with manufacturer’s instructions. Circulating RNA from plasma samples was obtained using miRNeasy Serum/Plasma Kit (Qiagen, Hilden, Germany), according to manufacturer’s protocol. RNA concentrations and purity ratios were ascertained using NanoDrop Lite spectrophotometer (NanoDrop Technologies, Wilmington, DE, USA) and RNA samples were stored at − 80 °C.

### MicroRNAs’ cDNA synthesis

The cDNA synthesis from FFPE tissues RNA was performed in a Veriti^®^ Thermal Cycler (Applied Biosystems, Foster City, CA, USA) using miRCURY LNA™ Universal RT microRNA PCR (Exiqon, Vedbaek, Denmark) following manufacturer’s instructions. Circulating RNA was reverse transcribed to cDNA using TaqMan Advanced miRNA cDNA synthesis kit (Applied Biosystems, Foster City, CA, USA). All cDNA samples were then stored at − 20 °C.

### Real-time quantification of microRNA

For the detection of cDNA derived from tissue samples, per each well, it was added: 5 μL of Xpert Fast SYBR (2X) (GRiSP, Porto, Portugal), 1 μL of miRNA specific primer mix (microRNA LNA™ PCR primer set, Exiqon, Vedbaek, Denmark), in accordance with manufacturer’s protocol, and 4 μL of previously diluted (20X) cDNA. For detection of cDNA derived from circulating miRNAs, per each well, it was added: 5 μL of Xpert Fast Probe (2X) (GRiSP, Porto, Portugal), 0.5 μL of TaqMan^®^ Advanced miRNA Assay (20X) (Applied Biosystems, Foster City, CA, USA) and 4.5 μL of diluted cDNA.

Quantitative real-time PCR (RT-qPCR) reactions were performed in 384-well plates. Each amplification reaction was performed in triplicate on a LightCycler 480 instrument (Roche Diagnostics, Manheim, Germany). Each plate also contained two negative template controls.

For the intercalating green dye chemistry, RT-qPCR protocol consisted of a denaturation step at 95 °C for 2 min, followed by 40 amplification cycles at 95 °C for 5 s and 60 °C for 20 s. Melting curve analysis was performed according to instrument’s manufacturer recommendations. For the probe-detection technology, RT-qPCR protocol consisted of a denaturation step at 95 °C for 3 min, followed by 45 amplification cycles at 95 °C for 10 s and 60 °C for 25 s.

SNORD38B was used as a reference gene for normalization as previously reported [16]. The relative miRNA expression in each tissue RNA sample was calculated using the 2^−ΔCT^ method: (Relative miRNA expression = 2^−ΔCt^, in which ΔCt = Ct _target miRNA_ − Ct_reference_) × 1000, whereas for liquid biopsies, relative miRNA expression was calculated using the formula: (Mean quantity of target miRNA/Mean quantity of SNORD38B) × 1000. Five serial 10 × dilutions of positive control were run in each plate to generate a standard curve. All plates had an efficiency between 90 and 100%. The target sequences of mature miRNAs analyzed are provided in Additional file 1: Table S1.

### Statistical analysis

Non-parametric Mann–Whitney U test and Kruskal–Wallis test, followed by Mann–Whitney U tests when appropriate, were used to ascertain the statistical significance of differences in continuous variables among two or more independent datasets, respectively. Bonferroni correction was applied to pairwise comparisons. Differences between paired samples were analyzed using the non-parametric Wilcoxon paired sample test. Fold changes for miRNA were calculated using the 2^−ΔΔCT^ method [19]. Spearman non-parametric test was performed to assess the correlation between continuous variables.

Receiver Operating Characteristic (ROC) curve was constructed and biomarker performance parameters (sensitivity, specificity, positive predictive value (PPV), negative predictive value (NPV) and accuracy) were calculated. The cut-off was established based on the highest value obtained in ROC curve analysis according to Youden’s J index [20, 21].

Statistical analysis was performed using SPSS software (SPSS Version 24.0, Chicago, IL) and two-tailed *p*-values were considered statistically significant when *p *< 0.05. Graphs were built using GraphPad 6 Prism (GraphPad Software, USA).

## Results

### Evaluation of miRNAs expression in FFPE BrC patients’ testing cohort

MiR-30b-5p, miR-30c-5p, miR-182-5p and miR-200b-3p expression levels were assessed in FFPE tissues from the testing cohort. Four of the 16 patients had multiple metastases at different sites (Additional file 1: Table S2). The time between diagnosis of the primary tumor and metastasis varied from 1.51 to 20.43 years (median 7.27 years).

**Fig. 1:**
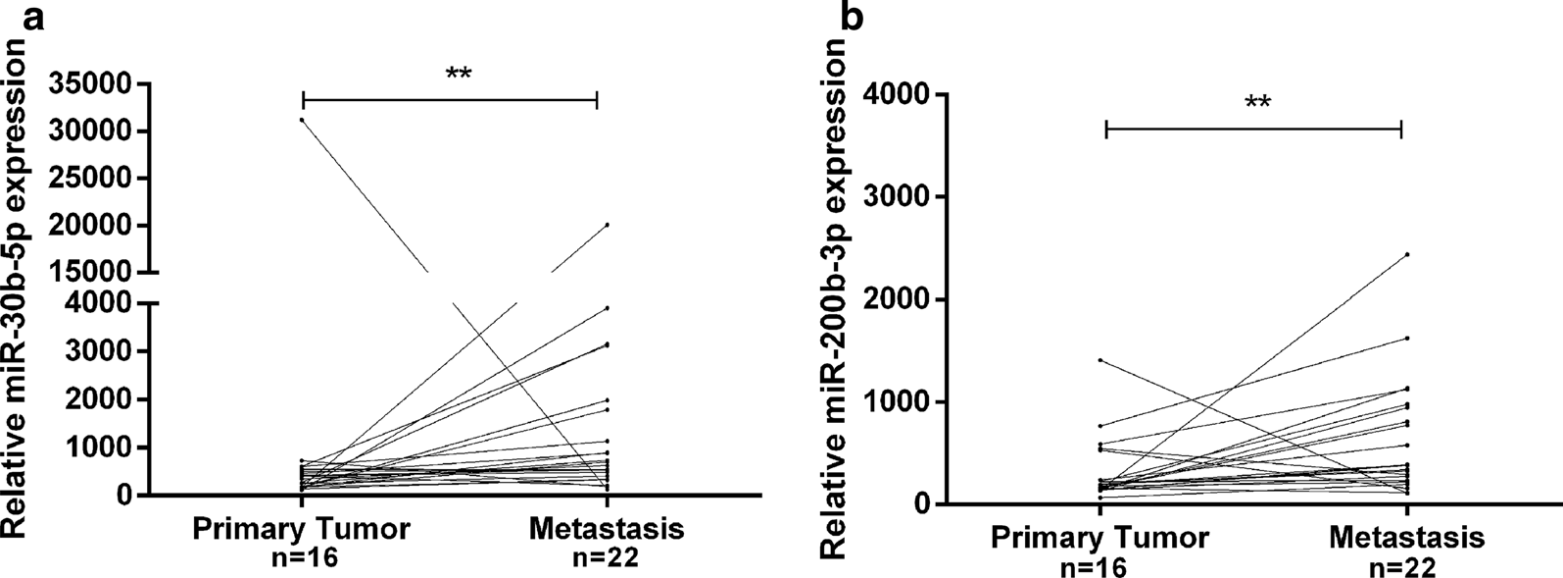
MiR-30b-5p **(a)** and miR-200b-3p **(b)** relative expression levels in primary tumors and the corresponding paired metastases. ** *p* value < 0.01 by non-parametric Wilcoxon paired sample test. Y-axis denotes 2^−ΔCT^ values multiplied by 1000

MiR-30b-5p and miR-200b-3p expression levels were significantly higher in metastatic lesions *versus* matched primary tumors (*p *= 0.007 and *p *= 0.009, respectively, Fig. 1a, b). Furthermore, miR-30b-5p and miR-200b-3p expression levels were significantly higher in metastatic lesions, in 10 of 16 patients and in 11 of 16 patients, respectively, both with a fold variation higher than 1. No significant expression differences were found between primary tumors and the corresponding metastatic lesions for the remain miRNAs.

### MiR-30b-5p and miR-200b-3p expression levels in cohort #1 and association with clinicopathological features

MiR-30b-5p and miR-200b-3p were further tested in a larger set of 175 tumor samples (validation cohort #1, Table 1 and Additional file 1: Table S3). Among the 93 available metastases, 63 were from bone, 17 from lung, 4 from brain and 9 were locoregional or from the contralateral breast. It should be noted that 10 patients had multiple metastases with different locations (Additional file 1: Table S3). Overall, the time elapsed between diagnosis of the primary tumor and metastasis varied from 0.15 to 18.98 years (median 6.63 years).

**Table 1:** Breast cancer patients’ clinicopathological data of the testing and validation cohort #1

Clinicopathological features	Testing cohort	Validation cohort #1
Patients (n)	16	82
Age median (range)	58 (35–78)	49 (28–76)
Molecular subtype^a^ (%)		
Luminal A-Like	4 (25.0)	19 (23.2)
Luminal B-Like	12 (75.0)	58 (70.7)
HER2-enriched	–	1 (1.2)
Basal-like/TNBC	–	4 (4.9)
Histological type (%)		
Invasive carcinoma of NST	13 (81.2)	73 (89.0)
Invasive lobular carcinoma	3 (18.8)	–
Other special subtype carcinoma	–	–
Mixed type carcinoma	–	9 (11.0)
Grade (%)		
G1	2 (12.5)	4 (4.9)
G2	4 (25.0)	43 (52.4)
G3	8 (50.0)	35 (42.7)
Gx	2 (12.5)	–
ER receptor *status* (%)		
Positive	16 (100.0)	77 (93.9)
Negative	–	5 (6.1)
PR receptor *status* (%)		
Positive	10 (62.5)	65 (79.3)
Negative	6 (37.5)	17 (20.7)
HER2 receptor *status* (%)		
Positive	3 (18.8)	15 (18.3)
Negative	13 (81.2)	67 (81.7)
T Stage (%)		
T1	4 (25.0)	20 (24.4)
T2	9 (56.2)	52 (63.4)
T3	1 (6.3)	5 (6.1)
T4	2 (12.5)	3 (3.7)
Tx	–	2 (2.4)
N Stage (%)		
N0	5 (31.2)	18 (22.0)
N1	7 (43.8)	33 (40.2)
N2	2 (12.5)	16 (19.5)
N3	2 (12.5)	13 (15.9)
Nx	–	2 (2.4)
Stage (%)		
I	3 (18.8)	10 (12.2)
II	8 (50.0)	34 (41.5)
III	5 (31.2)	24 (29.3)
IV	–	12 (14.6)
Not determined	–	2 (2.4)

*ER* estrogen receptor, *G* grade, *HER2* human epidermal growth factor receptor 2, *NST* no special type, *PR* progesterone receptor

^a^Assessed by immunohistochemistry

**Fig. 2:**
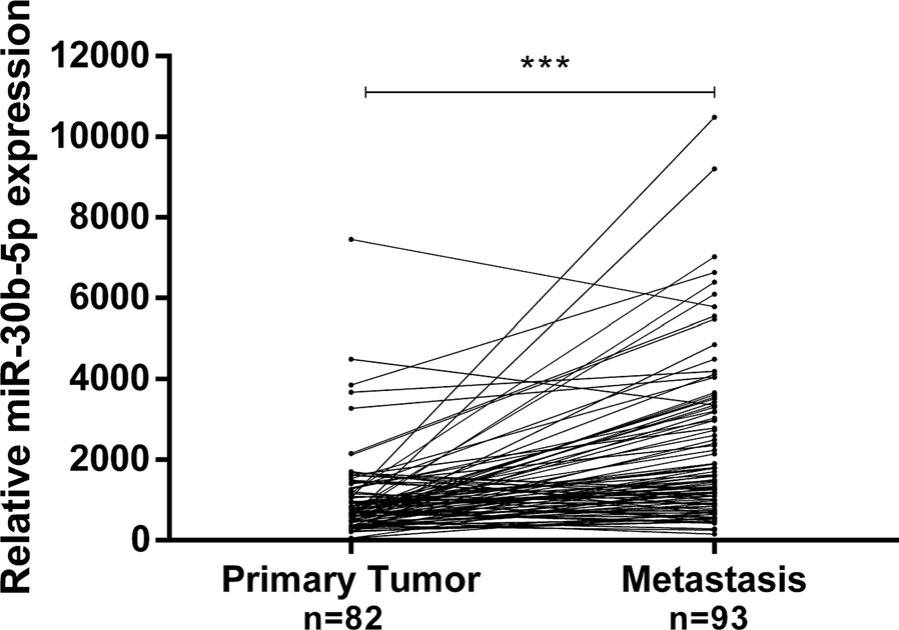
MiR-30b-5p relative expression levels in primary tumors and the corresponding matched metastases. *** *p*-value < 0.001 by non-parametric Wilcoxon paired sample test. Y-axis denotes 2^−ΔCT^ values multiplied by 1000

**Fig. 3:**
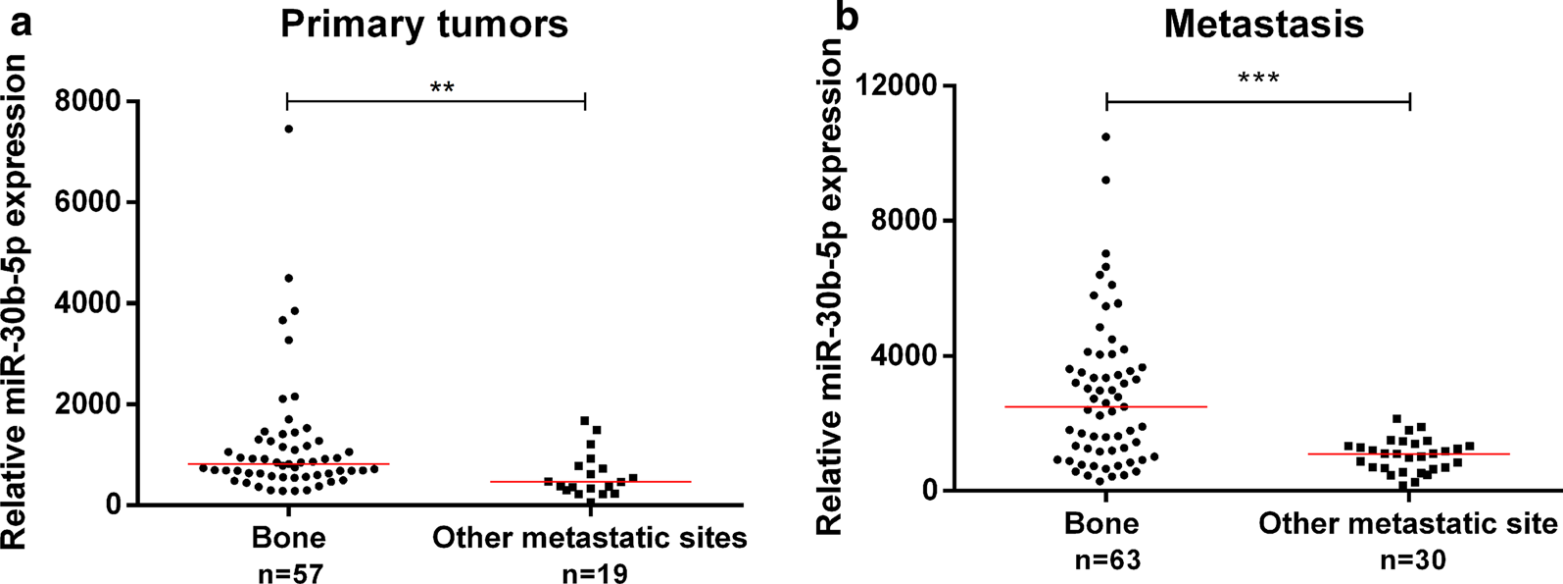
Scatter-plots of miR-30b-5p relative expression in primary tumors **(a)** and metastases **(b)**. ** *p*-value < 0.01 and *** *p*-value < 0.001 by non-parametric Mann–Whitney U test. Y-axis denotes 2^−ΔCT^ values multiplied by 1000

MiR-30b-5p expression levels were significantly higher in metastases than in primary tumors (*p *< 0.001, Fig. 2), confirming the findings obtained in the above-mentioned testing cohort. Interestingly, primary tumors that metastasized to bone disclosed significantly higher miR-30b-5p expression levels compared to all other primary tumors (*p *= 0.002, Fig. 3a). Moreover, bone metastases displayed significantly higher miR-30b-5p expression levels than all samples from other metastatic sites (*p *< 0.001, Fig. 3b).

Except for human epidermal growth factor 2 receptor (HER2) *status*, no statistically significant associations were found between miR-30b-5p expression levels and any of the clinicopathological parameters (age, histological type, grade, TNM staging, molecular subtype assessed by immunohistochemistry, estrogen receptor (ER) and progesterone receptor (PR) *status*). Indeed, HER2-negative tumors depicted significantly higher miR-30b-5p expression levels compared to HER2-positive BrC (*p *= 0.041).

The same analysis was performed for miR-200b-3p’s expression levels, however, no statistical differences were observed between primary tumors and matched metastases neither significant associations were found with clinicopathological parameters.

### Assessment of miR-30b-5p expression as prognostic biomarker in liquid biopsies (validation cohort #2)

A BrC patient cohort composed of 20 patients with localized BrC (stage I) and 25 patients with advanced BrC, comprising both locally advanced (n = 13) and metastatic BrC (n = 12) was used for validation of miR-30b-5p in liquid biopsies (validation cohort #2, Table 2). No significant differences were found for patients’ age between localized and advanced BrC (*p *= 0.417).

**Table 2:** Clinicopathological data of breast cancer patients of validation cohort #2

Clinicopathological features	Localized BrC	Advanced BrC
Patients (n)	20	25
Age median (range)	61 (39–71)	53 (35–82)
Molecular subtype^a^ (%)		
Luminal A-Like	7 (35.0)	2 (8.0)
Luminal B-Like	13 (65.0)	16 (64.0)
HER2-enriched	–	5 (20.0)
Basal-like	–	2 (8.0)
Histological type (%)		
Invasive carcinoma of NST	17 (85.0)	18 (72.0)
Invasive lobular carcinoma	2 (10.0)	4 (16.0)
Other special subtype carcinoma	–	2 (8.0)
Mixed type carcinoma	1 (5.0)	1 (4.0)
Grade (%)		
G1	4 (20.0)	–
G2	9 (45.0)	19 (76.0)
G3	7 (35.0)	6 (24.0)
ER receptor *status* (%)		
Positive	20 (100.0)	18 (72.0)
Negative	–	7 (28.0)
PR receptor *status* (%)		
Positive	20 (100.0)	14 (56.0)
Negative	–	11 (44.0)
HER2 receptor *status* (%)		
Positive	4 (20.0)	11 (44.0)
Negative	16 (80.0)	14 (56.0)
T Stage (%)		
T1	20 (100.0)	3 (12.0)
T2	–	11 (44.0)
T3	–	5 (20.0)
T4	–	6 (24.0)
N Stage (%)		
N0	20 (100.0)	–
N1	–	4 (16.0)
N2	–	–
N3	–	20 (80.0)
Nx	–	1 (4.0)
Stage (%)		
IA	20 (100.0)	n.a.
IIIC	n.a.	13 (52.0)
IV	n.a.	12 (48.0)

*BrC* breast cancer, *ER* estrogen receptor, *G* grade, *HER2* human epidermal growth factor receptor 2, *n.a.* not applicable, *NST* no special type, *PR* progesterone receptor

^a^Assessed by immunohistochemistry

**Fig. 4:**
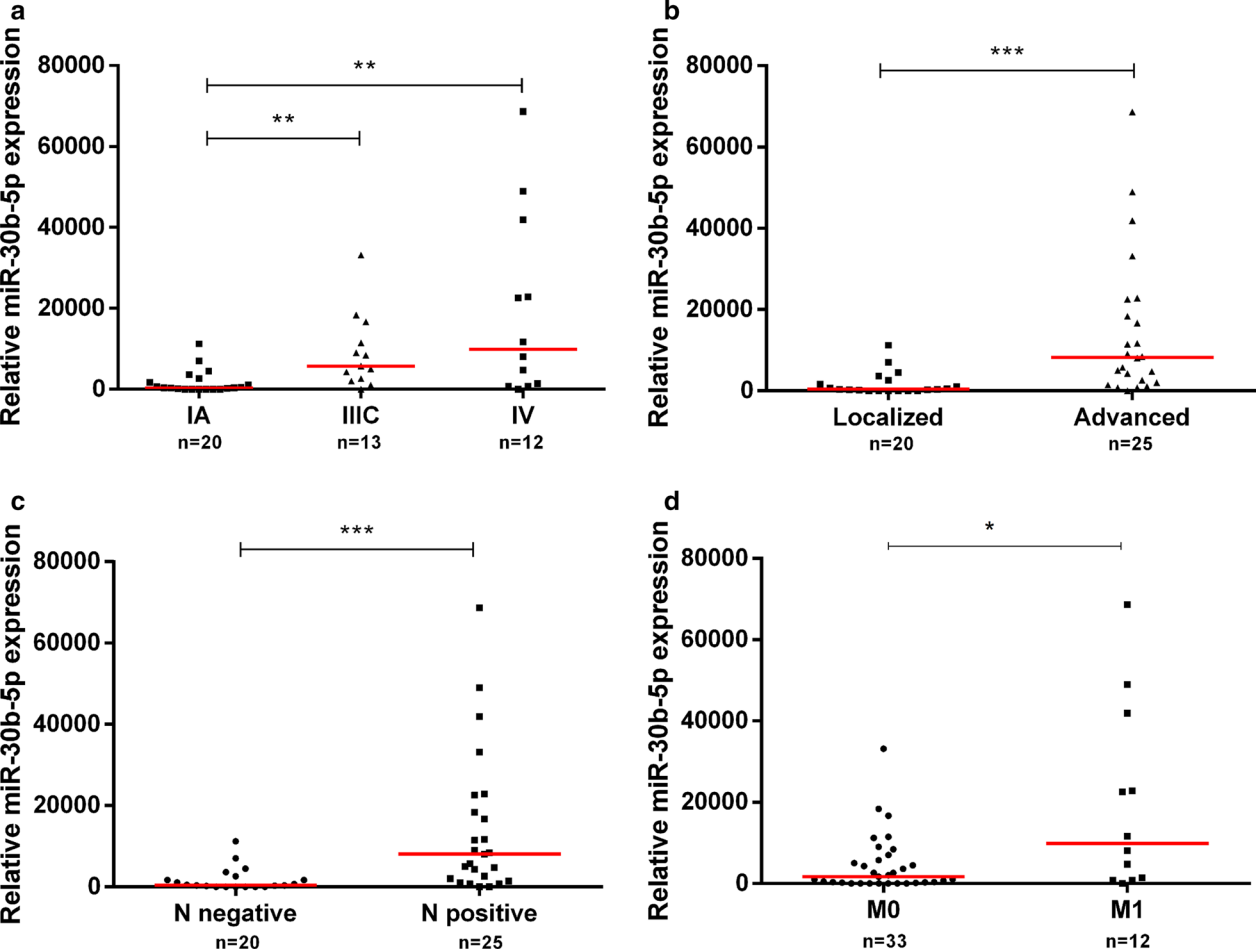
Scatter-plot of circulating miR-30b-5p relative expression according to stage **(a)**. ** *p*-value < 0.01 by non-parametric Kruskal–Wallis test. Y-axis denotes circulating miR-30b-5p relative expression multiplied by 1000. Scatter-plots of circulating miR-30b-5p relative expression in localized and advanced breast cancer **(b)** and according to N stage **(c)** and M stage **(d)**. * *p*-value < 0.05 and *** *p*-value < 0.001 by non-parametric Mann–Whitney U test. Y-axis denotes circulating miR-30b-5p relative expression multiplied by 1000

Remarkably, patients with locally advanced (stage III) and patients with metastatic BrC (stage IV) displayed higher circulating miR-30b-5p expression levels compared to localized BrC (*p *= 0.003 and *p *= 0.003, respectively, Fig. 4a). Moreover, advanced BrC group comprising patients with locally advanced and metastatic disease presented significantly higher circulating miR-30b-5p levels than patients with localized disease (*p *< 0.001; Fig. 4b).

Accordingly, circulating miR-30b-5p levels were significantly higher in patients with T2 tumors and grade 2 tumors (*p *= 0.018 and *p *= 0.012, respectively) and in patients with positive axillary lymph nodes and metastases at the time of diagnosis (*p *< 0.001, Fig. 4c and *p *= 0.015, Fig. 4d, respectively). No significant differences were observed for miR-30b-5p circulating levels and any of the clinicopathological parameters (age, histological type, molecular subtype assessed by immunohistochemistry, HER2, ER, and PR *status*).

**Fig. 5:**
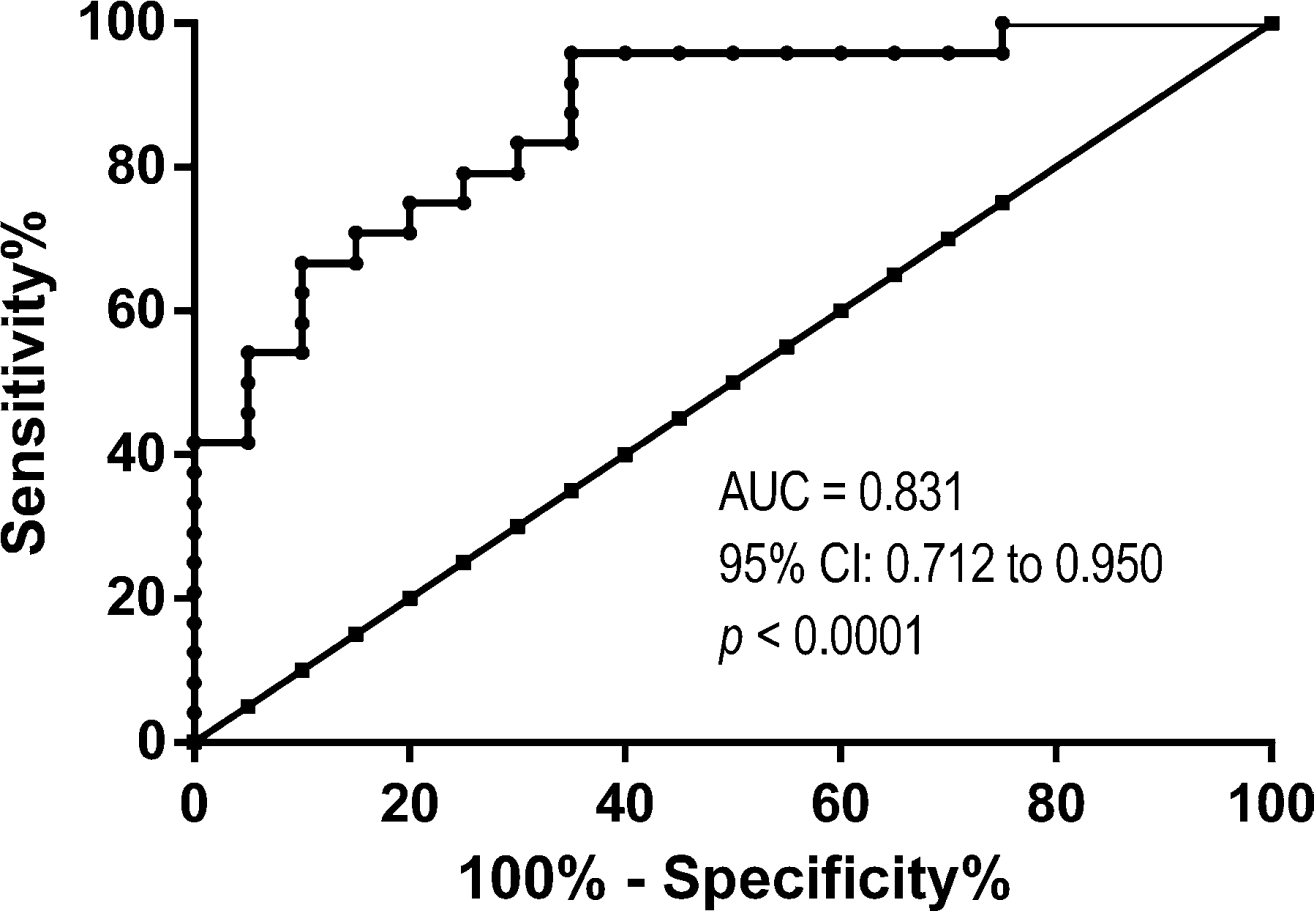
ROC curve analysis to evaluate the potential of miR-30b-5p as a biomarker for discriminate patients with advanced breast cancer from patients with localized breast cancer

ROC analysis revealed that circulating miR-30b-5p expression levels could discriminate advanced from localized BrC patients with an area under the curve (AUC) of 0.831 (95% CI 0.721–0.950). Using a cut-off value of 4611, circulating miR-30b-5p expression identified advanced disease with 88.9% sensitivity, 66.7% specificity, 75.6% accuracy, 64.0% PPV and 90.0% NPV (Fig. 5).

## Discussion

BrC remains the most common malignancy in women and a major cause of morbidity and mortality [1]. Although biological features are routinely used for BrC diagnosis and prognosis assessment, patients with similar clinicopathological features often show different clinical outcomes [22]. Therefore, identification of biomarkers providing more accurate prognostic information for BrC patients, complementing currently used parameters, will have a major impact. Consequently, assessment of specific miRNAs expression deregulation, which has been associated with several mechanisms underlying BrC aggressiveness, might be a potential source for biomarkers [7, 23]. Most studies addressing miRNAs expression and miRNA-target validation have been performed in cancer cell lines lacking tumor-host interactions [7, 24]. Thus, tissue analysis might allow for a broader insight into biologically and clinically relevant miRNAs which may serve as prognostic biomarkers.

In previous work, we reported a panel of miRNAs related to luminal BrC patients’ prognosis, suggesting these miRNAs as potential progression biomarkers [16]. Hence, we aimed to evaluate the biomarker potential of miR-30b-5p, miR-30c-5p, miR-182-5p and miR-200b-3p for progression/metastization in BrC.

In the testing cohort, only miR-30b-5p and miR-200b-3p expression were significantly higher in metastatic lesions compared to matched primary BrC tissues. Although increased miR-182-5p expression levels have previously been detected in paired metastatic lymph nodes when compared with primary tumors [15], we were not able to confirm the same in our testing set of BrC tissues. This might be due to the small number of samples, the restricted molecular subtype of the primary tumor and the variability of metastases localization.

MiR-30b-5p and miR-200b-3p were further evaluated in a larger set of tumor tissue samples from BrC patients (validation cohort #1). It should be recalled that the stability of miRNAs in FFPE tissues holds an enormous potential [25], especially in BrC patients in which late relapses frequently occur, as demonstrated in the testing cohort and validation cohort #1. Herein, miR-30b-5p expression levels were found to be significantly higher in metastatic lesions compared to matched primary BrC tissues in the validation cohort #1, despite the patients studied are mostly luminal, and the rather limited number of non-bone metastases. Although downregulation of miR-30f members and its role as tumor suppressor during BrC invasion and metastization have been previously reported [13, 14], miR-30b-5p upregulation in BrC metastatic tissue samples has not been described thus far. The role of miR-30b-5p remains controversial. On one hand, expression of miR-30b-5p and miR-30c-5p has been associated with increased cell viability and resistance to apoptosis [26], and miR-30b-5p was found to be upregulated in several cancers [27–29] and associated with metastases in melanoma [12]. On the other hand, miR-30b-5p was also associated with decreased migration and invasiveness in colorectal cancer [30, 31], and miR-30a was reported to be downregulated in primary nasopharyngeal carcinoma tissues but overexpressed in the corresponding metastases [32], although miR-30f members were shown to inhibit early steps of the metastatic process [33]. Our data clearly support an oncogenic role for miR-30b-5p in BrC.

Progression of solid malignancies is the result of a multi-step cascade in which tumor cells undergo widespread modifications to successfully migrate and colonize other organs. In this process, the dynamic ability to first undergo epithelial-to-mesenchymal transition (EMT) and subsequently mesenchymal-to-epithelial transition (MET) is an important feature of metastatic cells [34]. In support of the MET hypothesis, several studies have shown that metastatic lesions and the corresponding primary breast tumor have a similar epithelial nature [35, 36]. MiR-30b-5p modulation might be important in this plastic process. Downregulation of miR-30f members might lead to EMT initiation enabling cells to metastasize, while subsequent upregulation might be associated with MET, facilitating re-adaptation of the epithelial phenotype and colonization, crucial to develop macroscopic metastases. In fact, a recent study showed that downregulation of miR-30f members in primary BrC of patients without evidence of distant metastases was associated with poor relapse-free survival, which might be associated with the ability of decreased miR-30f levels to prompt EMT initiation. However, miR-30f expression levels were only evaluated in primary BrC tissue and were not assessed in metastatic lesions. Nevertheless, Kenny et al. [37] classified BrC cell lines into four morphological categories. BT-474 cell line, derived from a primary BrC, was included in Mass category characterized by tightly cohesive colonies with strong cell–cell adhesion, whereas MDA-MB-231, a metastatic cell line derived from a pleural effusion, was allocated to Stellate category characterized by limited cell–cell interactions and lacked *E*-*cadherin* expression, which are characteristics of EMT. Thus, these different phenotypes are in agreement with the previous hypothesis that decreased miR-30b-5p expression levels might lead to EMT initiation enabling cells with motility and invasive features. This might explain lower miR-30b-5p expression in MDA-MB-231 cells, and subsequent upregulation associated with MET, enabling re-adaptation of the epithelial phenotype, which was observed in BT-474 cells. Therefore, additional studies are needed to ascertain the miR-30b-5p functional role in BrC and the downstream transcriptional gene targets that might mediate distant metastases.

Knowledge of determining patterns of metastatic organ tropism might provide useful information for clinical evaluation of disease stage and to monitor progression. Hence, comparative analyses of miR-30b-5p expression according to the metastatic site were performed. Interestingly, bone metastases disclosed significantly higher miR-30b-5p expression levels compared to other metastases and, remarkably, primary BrC cases that metastasized to bone also displayed increased levels compared to those that did not. These results strongly suggest that not only miR-30b-5p plays a role in metastization, but it also predisposes tumor cells to home at specific organ sites, especially promoting bone colonization by tumor cells. Nevertheless, it should be recalled that miRNAs expression is highly context- and tissue-dependent, and thus, ideally, miRNA expression in normal tissues more prone to receive metastatic cells should also be assessed. We could then ascertain whether differential expression of miRNAs in the primary tumors *versus* metastatic tissues might be a consequence of their modulation in the metastatic microenvironment. Moreover, the mechanisms underlying tumor cell tropism to the bone and the extent to which metastatic cells miRNA’s profile differ according to their location may add valuable insights into disease development and clinical management. In fact, miR-30f members were reported to inhibit BrC bone metastases in an experimental model [38]. Nevertheless, these results were derived from triple negative BrC cell lines, which represent a (very) limited subset of BrC patients who do not commonly develop bone metastases. Nonetheless, it is well-recognized that BrC patients prone to develop bone metastases are mainly those which display luminal features [39, 40], and, thus this experimental model can hardly be considered representative of the clinically apparent heterogeneity.

Circulating miRNAs are stable in body fluids and their assessment might provide valuable diagnostic, prognostic and therapeutic prediction information, allowing for noninvasive testing and potential individual treatment optimization [7]. Recently, miR-30b-5p expression levels were shown to distinguish BrC patients from healthy controls in liquid biopsies [41], although miR-30b-5p expression levels have also been associated with aged [42]. Remarkably, we found that miR-30b-5p could discriminate patients with advanced BrC from those with localized BrC with high sensitivity, but modest specificity and overall accuracy, and no association with age was disclosed. Although the limited size of cohort #2 should be acknowledged, our results suggest that miR-30b-5p might identify, at diagnosis, patients who are more likely to endure disease progression. Particularly, circulating miR-30b-5p levels might provide a useful tool for early detection of BrC metastases, if these findings are further proven in a larger set of patients.

Several other miRNAs have been implicated in BrC invasion and metastasis [7]. MiR-10b was found highly expressed in tissue samples from patients with metastatic BrC [43] and, more recently, found to be significantly more expressed in tissues from patients with stage III and IV BrC compared to early-stage disease [44]. Moreover, circulating miR-10b combined with miR-373 might identify BrC lymph node metastasis with 72% sensitivity and 94.3% specificity [45], and miR-21 overexpression was significantly correlated with lymph node metastases, advanced clinical stage and poor prognosis [46]. Notwithstanding the tissue series size (n = 113), no stage IV patients were included in this study [46]. Similarly, circulating miR-21 discriminated stage IV BrC patients with visceral metastasis from those with stage I, II and III disease, with 86% specificity and 70% sensitivity [47]. Nonetheless, as far as we know, these metastasis-related miRNAs were only evaluated in primary BrC tissue and were not assessed in a larger tissue series of primary tumors and the corresponding metastatic lesions. Of note, in our study, circulating miR-30b-5p expression levels identified advanced disease with higher sensitivity, although with limited specificity.

## Conclusions

Our results indicate that miR-30b-5p is overexpressed in metastatic BrC, suggesting an important role in tumor dissemination. Interestingly, bone metastases and their correspondent primary tumors displayed higher miR-30b-5p expression levels, suggesting a role in modulation of metastatic organ tropism. Importantly, advanced BrC patients disclosed significantly higher circulating miR-30b-5p expression levels compared to patients with localized BrC, suggesting that circulating miR-30b-5p levels might identify BrC patients at higher risk of disease progression, providing a useful clinical tool for patient monitoring, entailing earlier and more effective treatment. Nonetheless, validation in larger multicentric cohorts is needed to further sustain our findings.

## Supplementary information

**Additional file 1: Table S1.** Specific target sequence of mature miRNAs tested. **Table S2.** Primary tumor and matched metastasis features from patients in the testing cohort. **Table S3.** Primary tumor and matched metastasis features from patients in the validation cohort #1.

## Data Availability

All data generated or analyzed during this study are included in this published article and its Additional information files.

## Abbreviations

AJCC: American Joint Committee on Cancer
AUC: area under the curve
BrC: breast cancer
EMT: epithelial-to-mesenchymal transition
ER: estrogen receptor
FFPE: formalin-fixed paraffin-embedded
HER2 receptor: human epidermal growth factor 2 receptor
MET: mesenchymal-to epithelial transition
MiR-200f members: MiR-200 family members
MiR-30f members: MiR-30 family members
MiRNAs: MicroRNAs
NPV: negative predictive value
PPV: positive predictive value
PR: progesterone receptor
ROC: receiver operating characteristic
RT-qPCR: quantitative real-time PCR
WHO: World Health Organization
