# Supplementary material for: Overexpression of circulating MiR-30b-5p identifies advanced breast cancer
Source: J Transl Med. 2019 Dec 30;17:435. doi: 10.1186/s12967-019-02193-y (PMC6936051; doi:10.1186/s12967-019-02193-y)
Supplement: Supplementary file 1 — Additional file 1: Table S1. Specific target sequence of mature miRNAs tested. Table S2. Primary tumor and matched metastasis features from patients in the testing cohort. Table S3. Primary tumor and matched metastasis features from patients in the validation cohort #1. [file 12967_2019_2193_MOESM1_ESM.docx]

Tables

Table S1 - Specific target sequence of mature miRNAs tested.

| **Gene** | **Target sequence** | **MirBase accession** |
| --- | --- | --- |
| **SNORD38B (hsa)** | UCUCAGUGAUGAAAACUUUGUCCAGUUCUGCUACUGACAGUAAGUGAAGAUAAAGUGUGUCUGAGGAGA | - |
| **hsa-miR-30b-5p** | UGUAAACAUCCUACACUCAGCU | MIMAT0000420 |
| **hsa-miR-30c-5p** | UGUAAACAUCCUACACUCUCAGC | MIMAT0000244 |
| **hsa-miR-182-5p** | UUUGGCAAUGGUAGAACUCACACU | MIMAT0000259 |
| **hsa-miR-200b-3p** | UAAUACUGCCUGGUAAUGAUGA | MIMAT0000318 |
| - Not applicable | | |

Table S2 - Primary tumor and matched metastasis features from patients in the testing cohort.

| **Patient number** | **Age at diagnosis** | **Molecular subtype**  **of primary tumor^1^** | **Metastasis localization** | **Time interval after**  **primary tumor (years)** |
| --- | --- | --- | --- | --- |
| **1** | 39 | Luminal B-Like | Lung | 20.43 |
| **2** | 60 | Luminal A-Like | Axillary lymph node | 16.07 |
| **3** | 36 | Luminal B-Like | Bone | 3.45 |
| **4** | 35 | Luminal B-Like | Liver | 11.05 |
| **5** | 74 | Luminal B-Like | Pleural | 11.75 |
| **6** | 64 | Luminal B-Like | Liver | 3.54 |
| **7** | 78 | Luminal B-Like | Breast Skin | 2.73 |
| **8** | 61 | Luminal B-Like | Bone | 2.76 |
| **9** | 43 | Luminal A-Like | Axillary lymph node | 11.68 |
| **10** | 55 | Luminal B-Like | Breast Skin | 6.55 |
| **11** | 51 | Luminal A-Like | Lung | 6.43 |
| **12** | 63 | Luminal B-Like | Pleural | 2.90 |
| **13** | 56 | Luminal B-Like | Breast skin | 3.48 |
|  |  |  | Axillary lymph node | 4.59 |
| **14** | 66 | Luminal A-Like | Mediastinum | 8.53 |
|  |  |  | Esophagus | 8.93 |
| **15** | 51 | Luminal B-Like | Contralateral breast | 6.44 |
|  |  |  | Axillary lymph node | 6.52 |
|  |  |  | Pleural | 11.02 |
|  |  |  | Contralateral breast skin | 11.39 |
| **16** | 60 | Luminal B-Like | Bone | 1.51 |
|  |  |  | Skin | 3.38 |
| ^1^ Assessed by immunohistochemistry | | | | |

**Table S3 -** Primary tumor and matched metastasis features from patients in the in validation cohort #1.

| **Patient number** | **Age at diagnosis** | **Molecular subtype**  **of primary tumor^1^** | **Metastasis localization** | **Time Interval after primary tumor (years)** |
| --- | --- | --- | --- | --- |
| **1** | 30 | Luminal B-Like | Brain | 10.07 |
| **2** | 37 | Basal-Like/TNBC | Brain | 1.2 |
| **3** | 36 | Luminal B-Like | Brain | 9.82 |
| **4** | 37 | Luminal B-Like | Brain | 10.45 |
|  |  |  | Lung | 10.4 |
| **5** | 28 | Luminal B-Like | Bone | 7.7 |
| **6** | 39 | Luminal B-Like | Bone | 9.95 |
|  |  |  | Lung | 11.26 |
| **7** | 32 | Luminal A-Like | Bone | 3.64 |
| **8** | 49 | Luminal A-Like | Bone | 13.08 |
| **9** | 51 | Luminal A-Like | Bone | 5.66 |
| **10** | 65 | Luminal B-Like | Bone | 8.80 |
| **11** | 64 | Luminal B-Like | Bone | 11.78 |
| **12** | 57 | Luminal B-Like | Bone | 2.47 |
| **13** | 44 | Luminal A-Like | Bone | 4.94 |
| **14** | 58 | Basal-Like/TNBC | Bone | 2.46 |
| **15** | 31 | Luminal B-Like | Bone | 0.15 |
| **16** | 57 | Luminal B-Like | Bone | - |
| **17** | 44 | Luminal B-Like | Bone | 9.52 |
| **18** | 76 | Luminal B-Like | Bone | 1.93 |
| **19** | 41 | Luminal B-Like | Bone | 3.41 |
| **20** | 71 | Luminal B-Like | Bone | 6.12 |
| **21** | 56 | Luminal B-Like | Bone | 6.66 |
| **22** | 46 | Luminal A-Like | Bone | 11.49 |
| **23** | 42 | Luminal B-Like | Bone | 1.94 |
| **24** | 56 | Luminal B-Like | Bone | - |
| **25** | 46 | Luminal A-Like | Bone | 7.67 |
| **26** | 36 | Luminal B-Like | Bone | 2.42 |
| **27** | 62 | Luminal A-Like | Bone | 6.47 |
| **28** | 46 | Luminal B-Like | Bone | 8.31 |
| **29** | 38 | Luminal B-Like | Bone | 3.84 |
| **30** | 33 | Luminal B-Like | Bone | 4.47 |
| **31** | 49 | Luminal B-Like | Bone | 6.61 |
|  |  |  | Locoregional | 7.05 |
|  |  |  | Contralateral Breast | 7.21 |
| **32** | 71 | Luminal B-Like | Bone | - |
| **33** | 43 | Luminal A-Like | Bone | 5.98 |
| **34** | 49 | Luminal A-Like | Bone | 12.61 |
|  |  |  | Contralateral Breast | 8.17 |
| **35** | 58 | Luminal B-Like | Bone | 4.99 |
| **36** | 40 | Luminal A-Like | Bone | 4.21 |
| **37** | 73 | Luminal B-Like | Bone | - |
| **38** | 58 | Luminal A-Like | Bone | 14.39 |
|  |  |  | Locoregional | - |
| **39** | 43 | Luminal A-Like | Bone | 3.42 |
| **40** | 64 | Luminal B-Like | Bone | 3.69 |
| **41** | 42 | Luminal A-Like | Bone | 4.83 |
| **42** | 47 | Luminal B-Like | Bone | 7.76 |
| **43** | 71 | Luminal A-Like | Bone | 3.44 |
| **44** | 51 | Luminal B-Like | Bone | 3.61 |
| **45** | 58 | Luminal B-Like | Bone | 5.50 |
| **46** | 40 | Luminal B-Like | Bone | 2.04 |
| **47** | 73 | Luminal A-Like | Bone | 4.71 |
| **48** | 61 | Luminal B-Like | Bone | 10.50 |
|  |  |  | Contralateral Breast | 3.78 |
| **49** | 47 | Luminal B-Like | Bone | 17.00 |
| **50** | 59 | Luminal B-Like | Bone | - |
| **51** | 43 | Luminal A-Like | Bone | 4.30 |
|  |  |  | Locoregional | 3.96 |
| **52** | 45 | Luminal B-Like | Bone | - |
| **53** | 33 | Luminal B-Like | Bone | 6.28 |
| **54** | 37 | Luminal A-Like | Bone | 7.07 |
| **55** | 53 | Luminal B-Like | Bone | - |
| **56** | 46 | Luminal B-Like | Bone | - |
| **57** | 69 | Luminal B-Like | Bone | 3.92 |
| **58** | 63 | Luminal B-Like | Bone | 11.72 |
| **59** | 65 | Luminal B-Like | Bone | - |
| **60** | 45 | Luminal B-Like | Bone | 3.18 |
| **61** | 61 | Luminal A-Like | Bone | 6.90 |
| **62** | 46 | Luminal B-Like | Bone | 7.93 |
| **63** | 53 | Luminal B-Like | Bone | 8.93 |
| **64** | 61 | Luminal B-Like | Bone | 1.92 |
| **65** | 32 | Luminal B-Like | Bone | 3.01 |
| **66** | 45 | Luminal B-Like | Bone | 4.81 |
| **67** | 56 | Basal-Like/TNBC | Bone | 8.38 |
| **68** | 43 | Luminal B-Like | Lung | 4.73 |
| **69** | 54 | HER2-enriched | Lung | 17.72 |
|  |  |  | Contralateral Breast | 13.19 |
| **70** | 70 | Luminal B-Like | Lung | 8.29 |
|  |  |  | Contralateral Breast | 9.70 |
| **71** | 75 | Luminal B-Like | Lung | - |
| **72** | 56 | Luminal B-Like | Lung | 18.98 |
| **73** | 35 | Luminal B-Like | Lung | 4.83 |
| **74** | 67 | Luminal B-Like | Lung | - |
| **75** | 51 | Luminal B-Like | Lung | 7.95 |
|  |  |  | Locoregional | 7.93 |
| **76** | 41 | Luminal B-Like | Lung | 9.43 |
| **77** | 48 | Luminal B-Like | Lung | 10.65 |
| **78** | 58 | Luminal B-Like | Lung | 6.15 |
| **79** | 74 | Luminal B-Like | Lung | 9.01 |
| **80** | 64 | Basal-Like/TNBC | Lung | 1.03 |
| **81** | 40 | Luminal A-Like | Lung | 12.13 |
| **82** | 54 | Luminal B-Like | Lung | - |
| ^1^ Assessed by immunohistochemistry   - Patients diagnosed with stage IV Breast Cancer | | | | |
